# Supplementary material for: Characterization of Mesothelin Glycosylation in Pancreatic Cancer: Decreased Core Fucosylated Glycoforms in Pancreatic Cancer Patients’ Sera
Source: Biomedicines. 2022 Aug 10;10(8):1942. doi: 10.3390/biomedicines10081942 (PMC9405996; doi:10.3390/biomedicines10081942)
Supplement: Supplementary file 1 [file biomedicines-10-01942-s001.zip › biomedicines-1782160-supplementary.pdf]

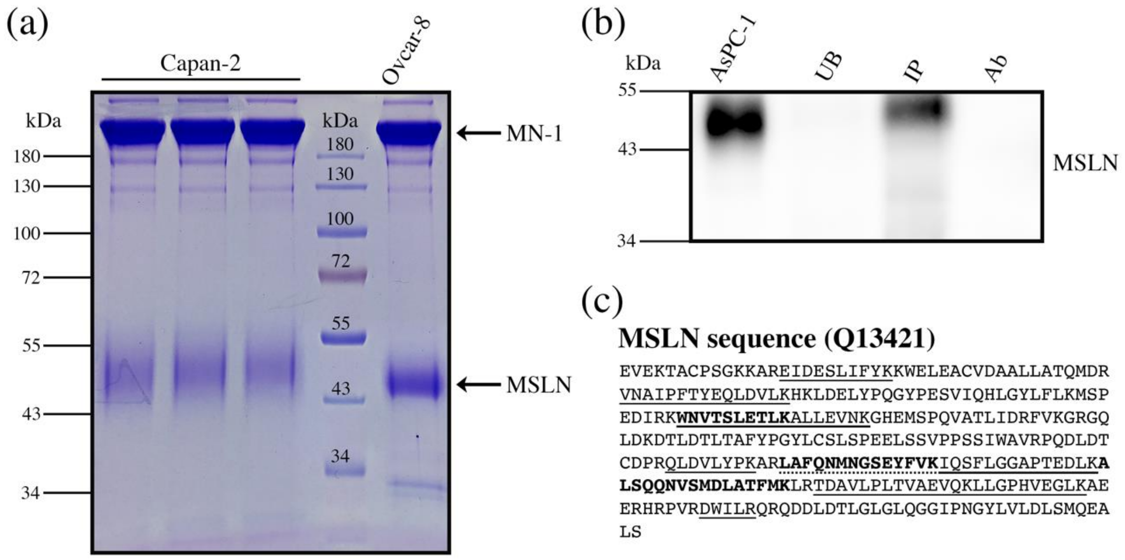

Figure S1. Mesothelin immunopurification with the anti-MSLN antibody (clone MN-1) used for subsequent glycosylation analyses. (**a**) Coomassie staining of immunopurified fractions from Capan-2 and Ovcar-8 cells conditioned media; (**b**) Anti-MSLN western blotting of obtained fractions during mesothelin immunopurification from AsPC-1 conditioned media. UB: unbound fraction, IP: purified fraction, Ab: MN-1 antibody; (**c**) Peptide mapping on MSLN immunopurified bands by MS (UPLC-ESI-QTof). Peptides containing a *N*-glycosylation motif in bold. Underlined peptides were identified in all cell lines. Dot-underlined peptide was only identified in Capan-2 bands.

Table S1. *N*-glycans identified in rMSLN for each exoglycosidase digestion. Structures arranged by retention time (GU), representation following symbol nomenclature for glycans (SNFG) guidelines and abundance expressed as the percentage among all assigned *N*-glycans in that profile. Sialic acid form: Neu5Ac, Neu5Gc.

Retention time (GU)	Structure	Representation	Abundance (%)
		**UNDIGESTED profile**	
9.25	A3G2Ga2	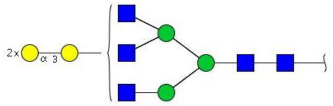	1.14
9.58	FA3G3Ga1	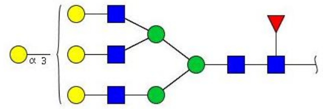	0.88
9.92	FA3F1G3Ga1	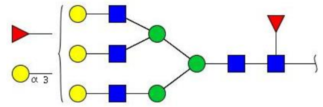	0.48
10.02	FA3F3G3	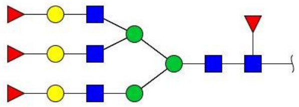	2.18
10.25	FA3F2G3Ga1	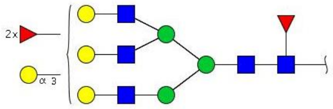	3.26
10.30	A3F2G3S1(6)	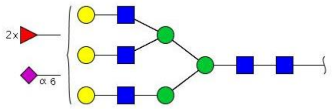	1.52
10.48	FA3G3Ga2	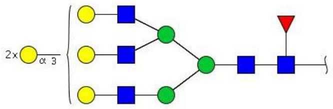	2.85
10.80	FA3F1G3Ga2	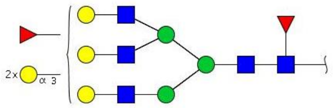	13.56
10.93	FA3G3S3(3,3,3)	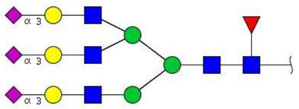	1.38
11.06	FA3G3S2(3,3)Ga1	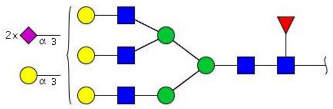	6.80
11.39	FA3G3Ga3	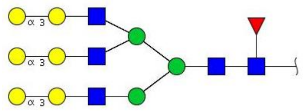	29.44
11.39	FA3F1G3S1(3)Ga1	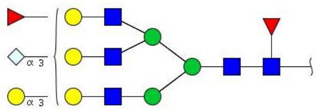	3.49
11.59	FA3F1G3S1(6)Ga1	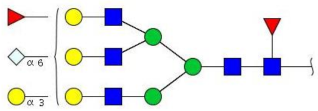	2.61

11.67	FA3G3S1(3)Ga2	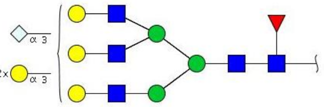	11.64
12.08	FA3G3S1(6)Ga2	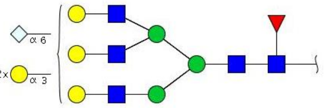	9.89
12.28	FA3G3S3(6,6,6)	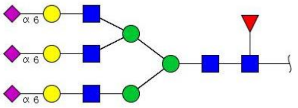	1.26
12.38	FA3G3S2(3,6)Ga1	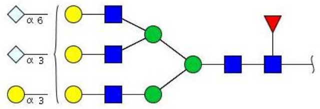	3.58
12.67	FA3G3Lac1Ga3	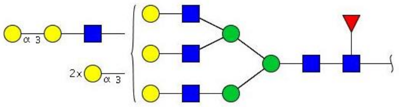	4.05
	**+NANI digestion profile**		
8.76	FA3G3	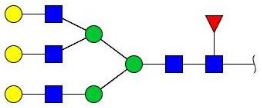	1.53
9.25	A3G2Ga2	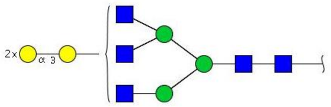	1.46
9.58	FA3G3Ga1	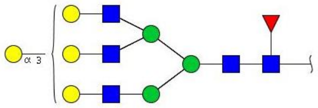	7.20
9.92	FA3F1G3Ga1	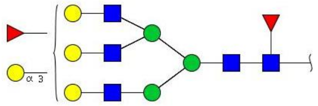	3.95
10.02	FA3F3G3	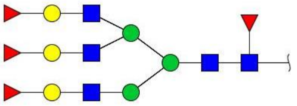	2.12
10.25	FA3F2G3Ga1	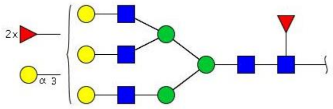	2.97
10.30	A3F2G3S1(6)	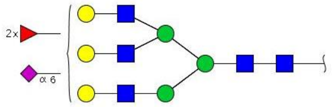	1.46
10.48	FA3G3Ga2	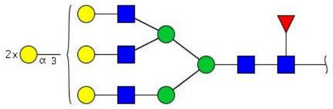	16.35
10.80	FA3F1G3Ga2	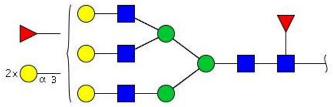	12.50
11.32	FA3G3S1(6)Ga1	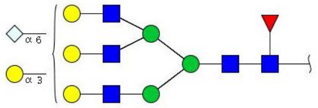	3.86

11.39	FA3G3Ga3	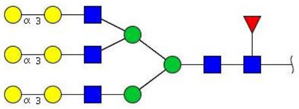	31.20
11.59	FA3F1G3S1(6)Ga1	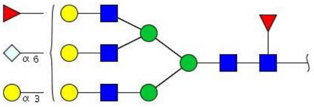	2.28
12.08	FA3G3S1(6)Ga2	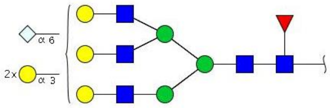	8.59
12.28	FA3G3S3(6,6,6)	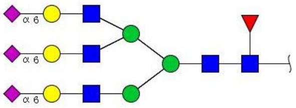	1.25
12.67	FA3G3Lac1Ga3	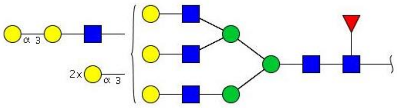	3.27
	**+ABS digestion profile**		
8.76	FA3G3	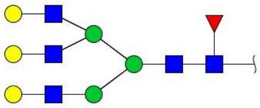	3.37
9.13	A3F2G3	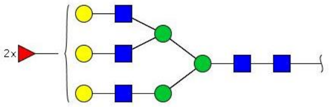	1.50
9.25	A3G2Ga2	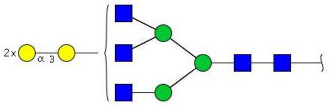	1.47
9.58	FA3G3Ga1	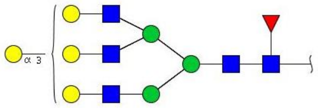	13.32
9.92	FA3F1G3Ga1	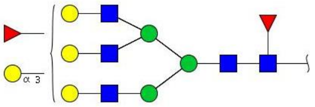	6.66
10.02	FA3F3G3	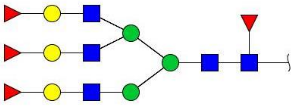	2.34
10.25	FA3F2G3Ga1	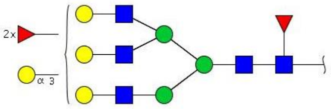	3.41
10.48	FA3G3Ga2	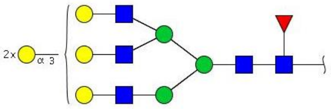	24.10
10.80	FA3F1G3Ga2	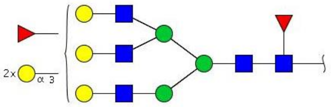	12.55
11.39	FA3G3Ga3	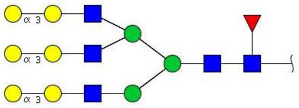	28.16

12.67	FA3G3Lac1Ga3	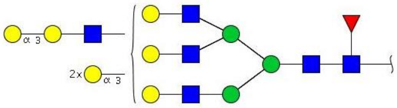	3.13
**+ABS+BTG digestion profile**			
6.20	FA3	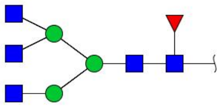	3.82
7.78	FA3G1Ga1	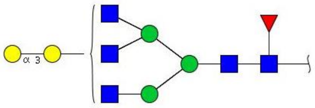	10.39
8.24	A3F2G2	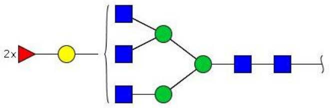	1.12
8.97	FA3F1G2Ga1	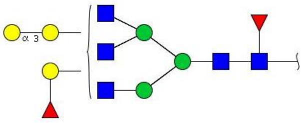	7.06
9.25	A3G2Ga2	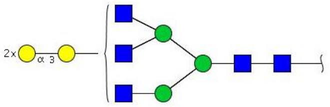	1.60
9.58	FA3G2Ga2	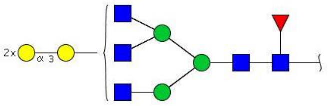	23.63
10.02	FA3F3G3	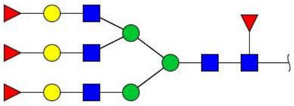	2.37
10.25	FA3F2G3Ga1	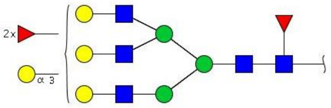	3.31
10.80	FA3F1G3Ga2	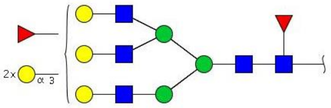	13.09
11.39	FA3G3Ga3	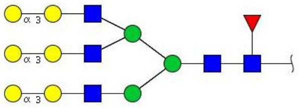	29.79
12.67	FA3G3Lac1Ga3	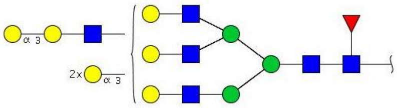	3.83
**+ABS+BTG+BKF digestion profile**			
5.79	A3	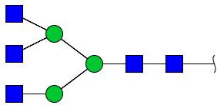	6.94
7.46	A3G1Ga1	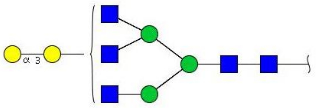	20.54

9.25	A3G2Ga2	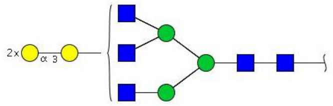	37.48
11.04	A3G3Ga3	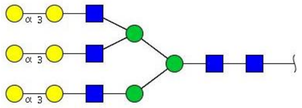	31.08
12.35	A3G3Lac1Ga3	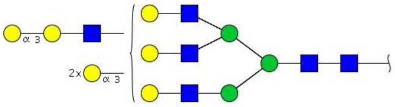	3.97
**+ABS+BTG+BKF+GUH digestion profile**			
4.30	M3	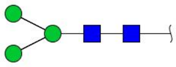	7.12
6.66	A1G1Ga1	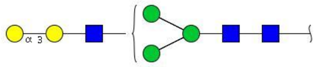	20.88
8.88	A2G2Ga2	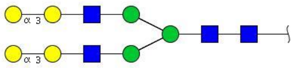	38.34
11.04	A3G3Ga3	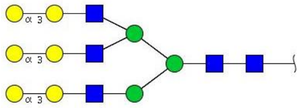	30.22
12.35	A3G3Lac1Ga3	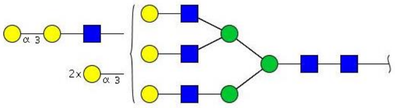	3.44
**+ABS+BTG+BKF+GUH+CBG digestion profile**			
4.30	M3	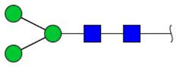	100.00

Table S2. *N*-glycans identified in mesothelin from Ovcar-8 conditioned media for each exoglycosidase digestion. Structures ordered by retention time (GU), representation following SNFG guidelines and abundance expressed as the percentage among all assigned *N*-glycans in that profile.

Retention time (GU)	Structure	Representation	Abundance (%)
**+NANI digestion profile**			
7.55	FA2G2	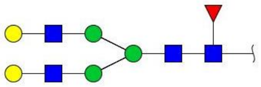	2.12
7.59	FA2BG2	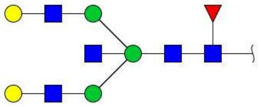	2.12
8.32	FA2F1G2	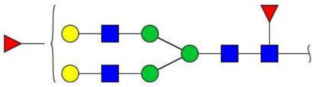 *fucose linked to antenna GlcNAc	2.95
8.32	A3G3	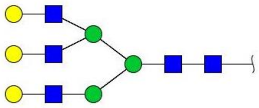	3.50
8.72	FA3G3	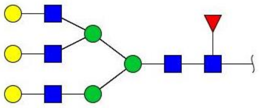	4.41
8.72	FA2G2S1(6)	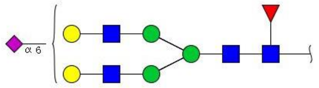	2.41
8.72	FA2BG2S1(6)	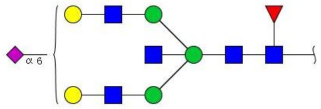	1.13
8.92	FA3BG3	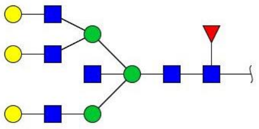	2.94
9.00	FA2F2G2	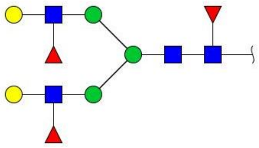	2.89
9.53	A4G4	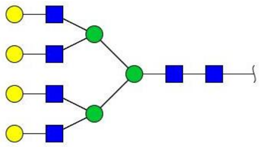	5.55

10.11	FA4G4	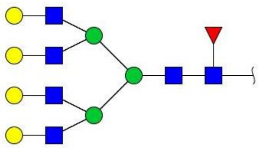	18.73
10.11	FA4BG4	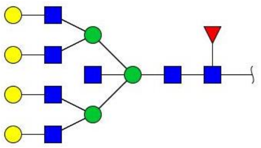	8.67
10.67	FA3F1G3Lac1	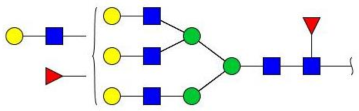 *fucose linked to antenna GlcNAc	5.38
10.83	FA3BF1G3Lac1	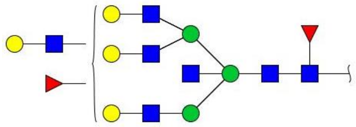 *fucose linked to antenna GlcNAc	2.06
10.83	A4F2G4	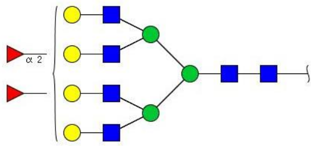 *α2 fucose linked to Gal *fucose linked to antenna GlcNAc	6.01
11.16	FA3G3Lac1S1(6)	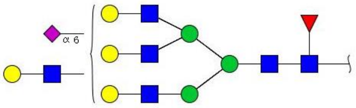	8.05
11.16	FA3BG3Lac1S1(6)	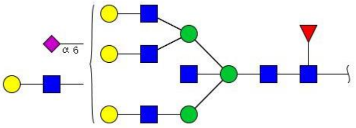	2.94
11.16	FA4G4S1(6)	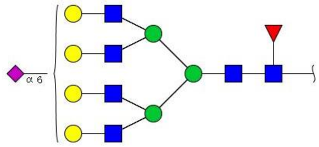	7.35
11.37	FA4G4Lac1	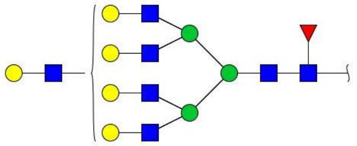	5.16
11.45	FA4BG4Lac1	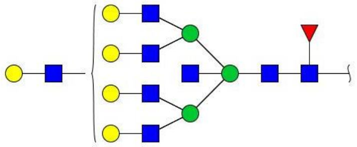	5.64

+ABS digestion profile			
7.55	FA2G2	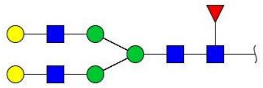	4.49
7.59	FA2BG2	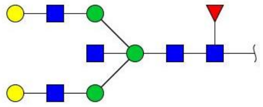	3.17
8.32	FA2F1G2	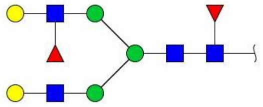	3.20
8.32	A3G3	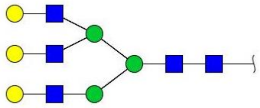	3.50
8.72	FA3G3	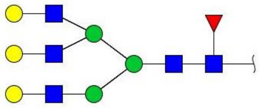	4.44
8.92	FA3BG3	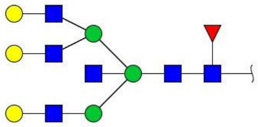	2.73
9.00	FA2F2G2	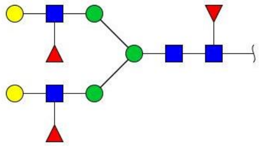	2.71
9.53	A4G4	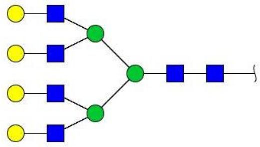	3.69
10.11	FA4G4	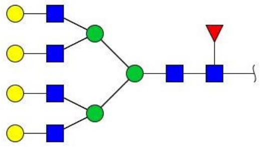	23.12
10.11	FA4BG4	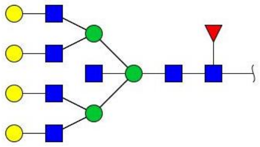	8.03
10.23	FA3G3Lac1	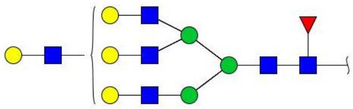	8.19

10.23	FA3BG3Lac1	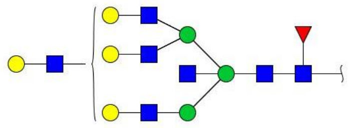	2.60
10.67	FA3F1G3Lac1	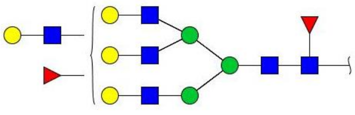<br>*fucose linked to antenna GlcNAc	5.28
10.83	FA3BF1G3Lac1	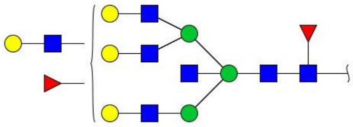<br>*fucose linked to antenna GlcNAc	2.01
10.83	A4F2G4	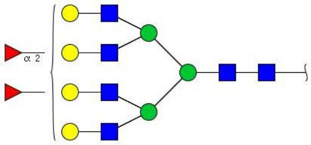<br>*α2 fucose linked to Gal<br>*fucose linked to antenna GlcNAc	7.76
11.37	FA4G4Lac1	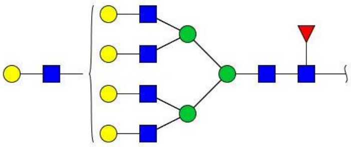	5.71
11.45	FA4BG4Lac1	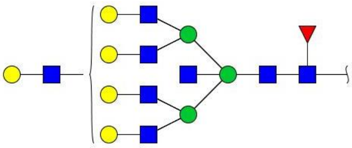	9.37
		**+ABS+BTG digestion profile**	
5.82	FA2	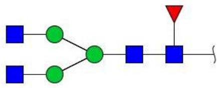	4.40
5.82	FA2B	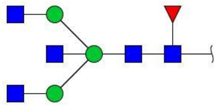	3.16
5.82	A3	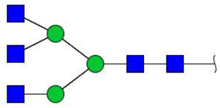	1.76
6.25	FA3	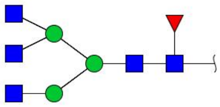	4.98
6.38	FA3B	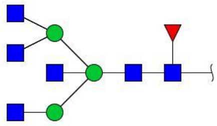	2.45

6.54	A4	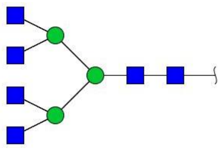	4.03
6.93	FA4	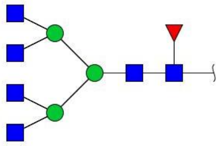	23.30
6.98	FA4B	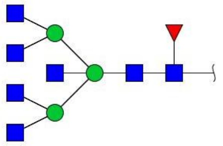	8.88
7.44	FA2F1G1	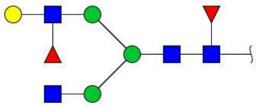	3.23
7.65	FA3G1GlcNAc1	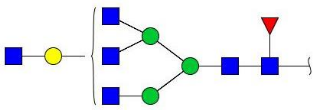	8.87
7.68	FA3BG1GlcNAc1	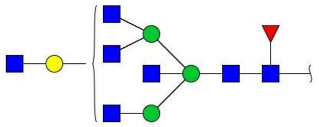	4.54
8.08	FA4G1GlcNAc1	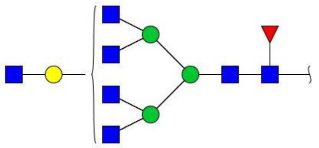	5.14
8.17	FA4BG1GlcNAc1	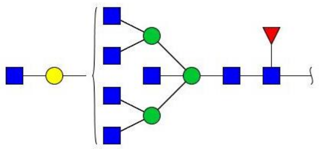	8.75
8.43	FA3F1G1GlcNAc1	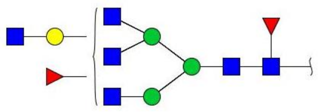	4.86
8.49	FA3BF1G1GlcNAc1	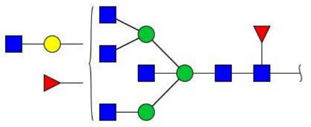	2.00
9.00	FA2F2G2	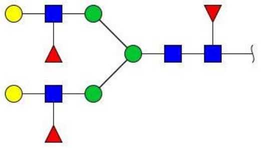	3.10

9.08	A4F2G2	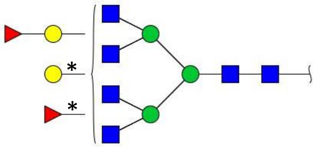 *Gal and *Fuc linked to same GlcNAc	6.55
**+ABS+BTG+AMF digestion profile**			
5.82	FA2	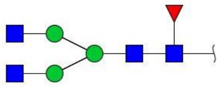	9.75
5.82	FA2B	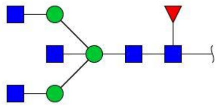	3.24
5.82	A3	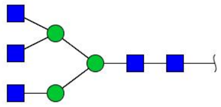	1.60
6.25	FA3	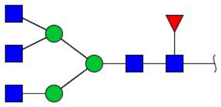	4.60
6.38	FA3B	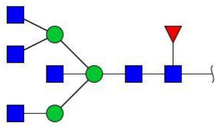	2.52
6.54	A4	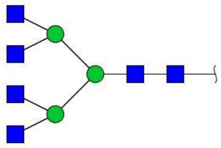	3.23
6.93	FA4	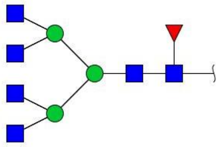	23.00
6.98	FA4B	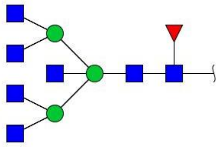	10.84
7.65	FA3G1GlcNAc1	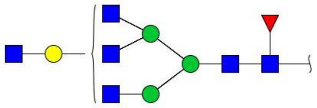	11.08
7.68	FA3BG1GlcNAc1	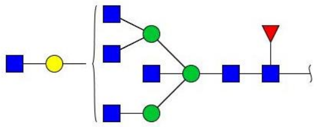	6.64

7.68	A4F1G1	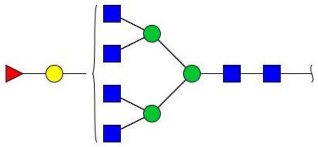	7.77
8.08	FA4G1GlcNAc1	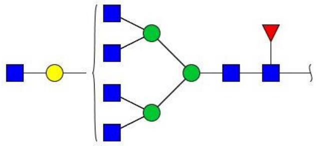	5.62
8.17	FA4BG1GlcNAc1	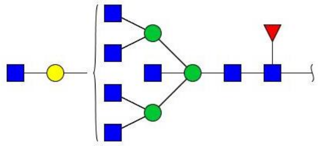	10.11
**+ABS+BTG+BKF digestion profile**			
5.36	A2	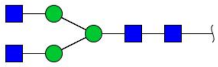	10.13
5.48	A2B	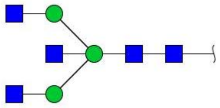	3.37
5.82	A3	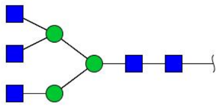	5.68
5.86	A3B	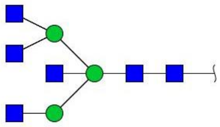	2.61
6.54	A4	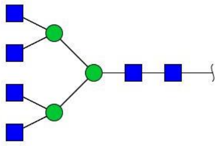	33.86
6.62	A4B	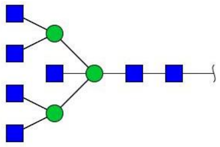	10.91
7.29	A3G1GlcNAc1	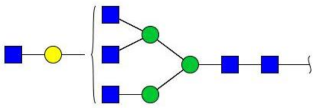	11.16
7.41	A3BG1GlcNAc1	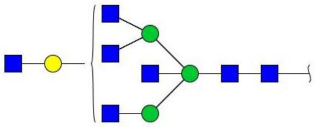	6.83

7.73	A4G1GlcNAc1	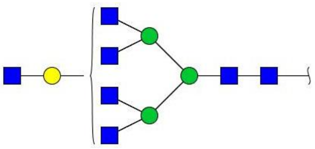	5.48
7.84	A4BG1GlcNAc1	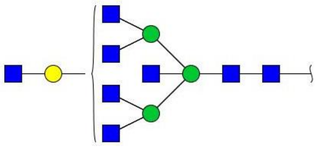	9.97
**+ABS+BTG+BKF+GUH digestion profile**			
4.25	M3	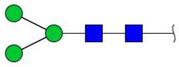	67.03
4.65	M3B	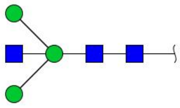	12.46
6.59	A4B	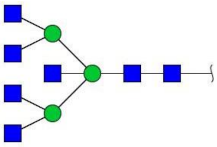	20.51

Table S3. *N*-glycans identified in mesothelin from PaC cell lines conditioned media after ABS digestion. Structures ordered by retention time (GU), representation following SNFG guidelines and abundance expressed as the percentage among all assigned *N*-glycans.

Retention time (GU)	Structure	Representation	Abundance (%)	
			Capan-2	AsPC-1
7.55	FA2G2	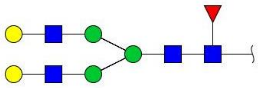	10.49	6.74
8.72	FA3G3	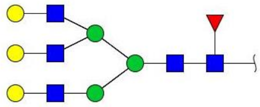	21.80	21.12
10.11	FA4G4	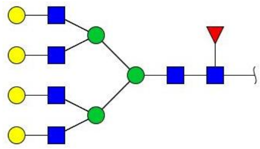	25.66	26.69
11.37	FA4G4Lac1	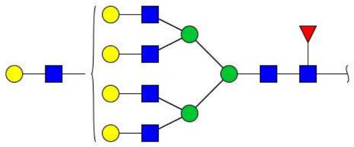	14.98	30.94
12.61	FA4G4Lac2	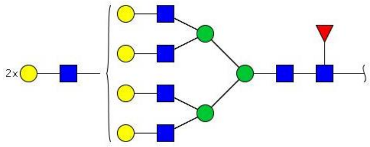	27.07	14.51
